# Inebriety

**Published:** 1911-05

**Authors:** 


### Inebriety.

De Lancy Carter, New York (*Medical Record,* February 11), regards every inebriate as an insane man who should be restrained in order to be cured and should be placed in a suitable hospital, with every resource for cure, until his self-respect is restored and he is physically and mentally able to stand alone. He should not be treated as a criminal and punished for what he can not help. Moral treatment has failed to cure the drunkard. Measures should be international. The patient must be where control and restraint are possible with physical restoration. Opportunities for physical labor in hygienic surroundings should be provided.—*Medical Herald.*

